# Surufatinib plus toripalimab in patients with advanced solid tumors: a single-arm, open-label, phase 1 trial

**DOI:** 10.1007/s00432-021-03898-8

**Published:** 2022-02-15

**Authors:** Yanshuo Cao, Ming Lu, Yu Sun, Jifang Gong, Jie Li, Zhihao Lu, Jian Li, Xiaotian Zhang, Yan Li, Zhi Peng, Jun Zhou, Xicheng Wang, Lin Shen

**Affiliations:** 1 Department of Gastrointestinal Oncology, Key Laboratory of Carcinogenesis and Translational Research (Ministry of Education), Peking University Cancer Hospital and Institute, Fu-Cheng Road 52, Hai-Dian District, Beijing, 100142 China; 2 Department of Pathology, Key Laboratory of Carcinogenesis and Translational Research (Ministry of Education), Peking University Cancer Hospital and Institute, Beijing, China; 3 Department of Early Drug Development Center, Key Laboratory of Carcinogenesis and Translational Research (Ministry of Education), Peking University Cancer Hospital and Institute, Beijing, China

**Keywords:** Surufatinib, Toripalimab, Advanced solid tumors, Safety, Preliminary efficacy

## Abstract

**Purpose:**

This phase 1 trial evaluated the safety, preliminary efficacy, and pharmacokinetics of surufatinib, a small molecular tyrosine kinase inhibitor, combined with toripalimab, a programmed cell death protein-1 antibody, in patients with advanced solid tumors.

**Methods:**

This is an open-label, dose-escalation and expansion study in patients with solid tumors who had failed standard therapies or had no effective treatment. In the dose-escalation stage, patients were treated with surufatinib, at dose levels of 200, 250, or 300 mg once daily (QD) in combination with toripalimab 240 mg, every 3 weeks (Q3W), to estimate maximum tolerated dose. Additional patients were enrolled in the dose expansion stage to further assess the efficacy, safety, and pharmacokinetics profile. Recommended phase 2 dose (RP2D) was determined based on the safety, tolerability, and preliminary efficacy from dose-escalation and expansion stages.

**Results:**

From Feb 14, 2019 to Dec 20, 2020, 33 patients were screened, of which 30 patients were enrolled. One patient in the 300 mg cohort experienced dose limited toxicity, a grade 3 hyperthyroidism. The most frequent treatment-related adverse events of grade ≥ 3 were hypertension (20.0%), transaminases increased (13.3%), and blood bilirubin increased (13.3%). No treatment-related death or treatment discontinuation was identified. The RP2D was determined to be surufatinib 250 mg QD plus toripalimab 240 mg Q3W. Objective response rate was 24.1% (95% confidence interval 10.3‒43.5%) in this study.

**Conclusions:**

Surufatinib plus toripalimab was well tolerated, with no unexpected safety signals, and showed preliminary anti-tumor activity in patients with advanced solid tumors.

**Trial registration:**

Clinicaltrials.gov Identifier: NCT03879057; registration date: March 18, 2019.

**Supplementary Information:**

The online version contains supplementary material available at 10.1007/s00432-021-03898-8.

## Introduction

During the past several years, immune checkpoint inhibitors (ICIs), such as antibodies against programed cell death protein-1 (PD-1) and programed cell death ligand-1 (PD-L1), have shown impressive clinical benefits in the treatment of solid tumors (Topalian et al. 2012; Ninomiya and Hotta 2018; Socinski et al. 2018; Robert et al. 2015). Although PD-1/PD-L1 antibodies have shown efficacy in patients with microsatellite instability-high (MSI-H) or mismatch repair-deficient (dMMR) status, efficacy of single-agent PD-1/PD-L1 inhibitors in treatment of patients harboring microsatellite stable/mismatch repair proficient (MSS/pMMR) seems to be less characterized (Oliveira et al. 2019; Abida et al. 2019; Le et al. 2015).

Attempts at increasing efficacy have been made using different combinations with ICIs. Preclinical evidence suggests that the combination of vascular endothelial growth factor/vascular endothelial growth factor receptor (VEGF/VEGFR) inhibitors and PD-1/PD-L1 inhibitors could lead to an increase in anti-tumor activity, by provoking T-cell function and modulating suppressive immune cells as well as the stroma, in the tumor microenvironment (Gunturi and McDermott 2014; Sharma and Allison 2015). The use of VEGF/VEGFR therapy could promote the extravasation and migration of immune inflammatory cells, thus enhancing the immune inflammatory response after the application of immunotherapy drugs, increasing the drugs’ activity against tumor cells and thereby improving the efficacy of immunotherapy drugs (Manegold et al. 2017). This notion is supported by mounting evidence in recent years, demonstrating the clinical benefits of VEGF/VEGFR therapy combined with immunotherapy for patients with advanced solid tumors (Rini et al. 2019; Herbst et al. 2019; Makker et al. 2019; Mo et al. 2021; Atkins et al. 2018; Powles et al. 2020).

In particular, the combination of VEGF/VEGFR inhibitor with an inhibitor of PD-1 has appeared to be effective for treatment of a number of different solid tumors. Studies using lenvatinib, a VEGFR inhibitor, plus pembrolizumab, an antibody against PD-1, elicited objective response rates (ORRs) of 70%, 53%, and 36.0% in patients with renal clear cell carcinoma, advanced endometrial cancer and unresectable hepatocellular carcinoma, etc. (Mo et al. 2021; Finn et al. 2020). In addition, recent results of the REGONIVO study, another combination of VEGF/VEGFR and PD-1 inhibitors, regorafenib and nivolumab, respectively, showed promising efficacy in the treatment of MSS gastric cancer and colorectal cancer (Fukuoka et al. 2020).

Surufatinib is a small molecule kinase inhibitor that primarily acts on VEGFR 1, 2, 3, fibroblast growth factor receptor 1 (FGFR 1), and colony-stimulating factor 1 receptor (CSF-1R). Surufatinib has demonstrated clinical activity in several solid tumors, especially in neuroendocrine tumor (NET) (Xu et al. 2019; Chen et al. 2020). Toripalimab is a recombinant humanized monoclonal immunoglobulin G4 (IgG4) antibody with a hinge S228P mutation that specifically targets PD-1, which has also shown efficacy in the treatment of solid tumors (Wang et al. 2019; Yang et al. 2020; Keam 2019). Preclinical studies have demonstrated that surufatinib decreased M2 tumor-associated macrophages (TAMs) and increased M1 TAMs, and this immune-modulation effect might result in enhanced anti-tumor effect when surufatinib is combined with anti-PD-1/PD-L1 antibody (Zhou et al. 2017). Herein, we report the results of a phase 1 study of surufatinib combined with toripalimab in the treatment of patients with advanced solid tumors.

## Materials and methods

### Study design and patients

This study is an open-label, dose-escalation, and dose-expansion trial conducted in Peking University Cancer Hospital, Beijing, China. All patients enrolled had unresectable or metastatic advanced solid tumors that were histologically or cytologically confirmed. Patients were 18‒75 years old, had failed standard treatment (due to disease progression or intolerable toxicity or side effects during the treatment), or had not responded to currently available therapies. Enrolled patients had physical performance score of 0–1 [Eastern Cooperative Oncology Group performance status (ECOG PS) score]. Patients with hepatocellular carcinoma had Child–Pugh A or B7 hepatic function. Patients also had measurable target lesions [per Response Evaluation Criteria in Solid Tumors (RECIST) version 1.1], adequate bone marrow function and liver function, an expected survival time of more than 12 weeks. Patients who experienced unrecovered anti-tumor treatment-related toxicity or had other malignant diagnosis, central nervous system (CNS), or brain metastases were excluded. Detailed inclusion and exclusion criteria are available in Supplementary Table S1.

This clinical trial was approved by Ethics Committee of Peking University Cancer Hospital and was conducted strictly in accordance with the Declaration of Helsinki and Good Clinical Practice. All patients provided written informed consent form (ICF) before enrollment. The study was registered at ClinicalTrials.gov with registration number NCT03879057.

### Treatment and assessment

All patients enrolled received the study treatment in 3-week cycles. In the dose-escalation stage, a modified “3 + 3” design was applied. Patients were assigned to one of the three surufatinib dose cohorts and received surufatinib 200, 250, or 300 mg orally once daily (QD, defined as 200 mg cohort, 250 mg cohort, and 300 mg cohort, respectively) in combination with a fixed dose of toripalimab (240 mg intravenously) every 3 weeks (Q3W) to evaluate the maximum tolerated dose (MTD). Dose-limited toxicity (DLT) assessment was performed within 28 days of the first dose. In the dose expansion stage, 3–6 patients were additionally enrolled into the 3 dose cohorts to further assess the efficacy, safety, and pharmacokinetic (PK) profile. Recommended phase 2 dose (RP2D) was determined based on the safety, tolerability, and preliminary efficacy from dose-escalation and expansion stage.

All patients received treatment until the first occurrence of disease progression, death, intolerable toxicity, or completion of the study. The dose of surufatinib could be adjusted according to the dose adjustment principle; however, dose reduction was not allowed for toripalimab.

Tumor assessments (per RECIST v1.1) were performed by investigators once every 6 weeks (± 7 days) from administration of the first dose, and once every 12 weeks (± 7 days) after 48 weeks of the treatment until disease progression, death, intolerable toxicity, or fulfillment of other criteria for terminating the study treatment, whichever occurred first. Patients were followed every 12 weeks (± 7 days) after discontinuation of the treatment to record their survival status until death, lost to follow up, or withdrawal of informed consent, whichever occurred first. All complete responses (CRs)/partial responses (PRs) were confirmed by a consecutive tumor assessment at least 4 weeks after the initial assessment of CR/PR.

Blood samples were collected for PK evaluation at multiple time points on Cycle 1 Day 1, Cycle 2 Day 1, and Cycle 3 Day 1 for surufatinib and toripalimab, respectively.

For patients who signed an optional biomarker ICF, additional tumor samples were collected to evaluate the correlation between the efficacy of surufatinib and/or toripalimab and PD-L1 expression. PD-L1 expression was detected by immunohistochemistry staining with SP263 antibody using a Ventana (Tucson, AZ) autostainer (Tsao et al. 2018). Positive PD-L1 expression was defined as the presence of membrane staining of any intensity in ≥ 1% tumor cells or in tumor-infiltrating immune cells in tumor area occupied by tumor cells, intratumoral cells, and peritumoral stroma.

The primary objective was to evaluate the safety and tolerability of surufatinib combined with toripalimab in patients with advanced solid tumors, and to determine DLT, MTD, and RP2D.

The secondary objectives were to evaluate the preliminary efficacy (per RECIST v1.1) and PK profile of surufatinib, combined with toripalimab, in patients with advanced solid tumors.

The exploratory objective was to evaluate potential biomarkers that predict the efficacy of surufatinib and/or toripalimab.

### Statistical analysis

DLT events were based on the DLT evaluable population, including patients who received at least 1 cycle of treatment and completed all safety assessments within the DLT assessment window or discontinued early due to DLT in the dose-escalation stage. All safety analyses, progression-free survival (PFS), and overall survival (OS) statistics were based on the safety population, including patients who received at least 1 dose of any study drug. All efficacy endpoints, excluding PFS and OS, were analyzed based on the efficacy evaluable population, including all patients who received at least 1 dose of any study drug and had tumor assessment at baseline and post drug administration prior to initiation of new anti-cancer therapies. The PK analysis was based on the PK population, which included all patients who received at least one dose of the study drug, had at least one PK sample collected and analyzed, and had no protocol deviation that might affect the PK data.

Two-sided 95% confidence interval (CI) of ORR and DCR were computed using the Exact (Clopper–Pearson) method. For time-to-event variables [PFS, OS, DoR (duration of response) and TTR (time to response)], median event time as well as the corresponding 95% CI were estimated using Kaplan–Meier method. All statistical analyses were performed using SAS 9.4 software. Adverse events (AEs) were graded by NCI CTCAE (Common Terminology Criteria for Adverse Events) v4.03, and coded using the Medical Dictionary for Regulatory Activities (MedDRA) v23.1. Treatment-related AEs (TRAEs) referred to AEs related to surufatinib or toripalimab, which were judged by the investigators. PK parameters were calculated by non-compartmental analysis using Phoenix WinNonlin v8.0.

## Results

From Feb 24, 2019 to Dec 20, 2020 (cut-off date), 33 patients were screened, and 30 were enrolled, including 6 in 200 mg cohort, 12 in 250 mg cohort, and 12 in 300 mg cohort, respectively. By the data cutoff, 6 patients (20.0%) were still on study treatment, including 5/12 patients (41.7%) in the 250 mg cohort, and 1/12 patients (8.3%) in the 300 mg cohort. Twenty-four of the 30 patients (80.0%) had discontinued study treatment at cut-off date; most of these study treatment discontinuations were due to disease progression (19 patients).

### Patient characteristics

Demographic and baseline characteristics are shown in Table 1. Most enrolled patients [24/30 patients (80.0%)] were male, and all patients (100%) presented with stage IV tumors at screening. Cancer types included neuroendocrine neoplasm [NEN; 22/30 patients (73.3%)], colorectal cancer [CRC; 4/30 patients (13.3%)], gastric adenocarcinoma [GC; 2/30 patients (6.7%)], esophageal squamous cell carcinoma [EC; 1/30 patients (1.3%)], and metastatic squamous cell carcinoma [1/30 patients (3.3%)]. The majority of enrolled NEN patients had neuroendocrine carcinoma [NEC; 14/30 patients (46.7%)], and the remaining 8 patients had NET (4 patients with NET G2 and 4 patients with NET G3).

**Table 1:** Patient demographics and baseline characteristics (safety population)

	Surufatinib 200 mg QD + toripalimab 240 mg Q3W (*n* = 6)	Surufatinib 250 mg QD + toripalimab 240 mg Q3W (*n* = 12)	Surufatinib 300 mg QD + toripalimab 240 mg Q3W (*n* = 12)	Total (*n* = 30)
Age (years)				
Mean (standard deviation)	55.2 (13.59)	55.8 (13.98)	59.2 (7.61)	57.0 (11.45)
Median	57.0	61.5	61.5	61.0
Min, max	36, 74	30, 71	45, 68	30, 74
Sex, *n* (%)				
Male	5 (83.3)	10 (83.3)	9 (75.0)	24 (80.0)
Female	1 (16.7)	2 (16.7)	3 (25.0)	6 (20.0)
Race, *n* (%)				
Asian	6 (100.0)	12 (100.0)	12 (100.0)	30 (100.0)
BMI (kg/m^2^)				
Mean (standard deviation)	20.83 (3.816)	25.46 (4.998)	21.93 (2.918)	23.12 (4.375)
Median	20.70	24.50	21.60	22.30
Min, max	15.4, 25.3	18.3, 36.9	16.3, 27.7	15.4, 36.9
Baseline ECOG PS, *n* (%)				
0	2 (33.3)	3 (25.0)	6 (50.0)	11 (36.7)
1	4 (66.7)	9 (75.0)	6 (50.0)	19 (63.3)
Cancer type, *n* (%)				
CRC	0	3 (25.0)	1 (8.3)	4 (13.3)
EC	0	1 (8.3)	0	1 (3.3)
GC	0	0	2 (16.7)	2 (6.7)
Metastatic squamous cell carcinoma	0	1 (8.3)	0	1 (3.3)
NEN	6 (100.0)	7 (58.3)	9 (75.0)	22 (73.3)
NEC	3 (50.0)	5 (41.7)	6 (50.0)	14 (46.7)
NET G2	0	1 (8.3)	3 (25.0)	4 (13.3)
NET G3	3 (50.0)	1 (8.3)	0	4 (13.3)
Site of primary tumor^a^, *n* (%)				
Colon	0	0	1 (11.1)	1 (4.5)
Stomach/gastroesophageal junction	0 (0.0)	1 (14.3)	1 (11.1)	2 (9.1)
Lung	0	1 (14.3)	0	1 (4.5)
Pancreas	2 (33.3)	1 (14.3)	1 (11.1)	4 (18.2)
Rectum	2 (33.3)	0	2 (22.2)	4 (18.2)
Stomach	2 (33.3)	2 (28.6)	0	4 (18.2)
Other	0	1 (14.3)	3 (33.3)	4 (18.2)
Tumor stage at screening, *n* (%)				
IV	6 (100.0)	12 (100.0)	12(100)	30 (100)

*BMI* body mass index, *ECOG* Eastern Cooperative Oncology Group, *PS* performance status, *CRC* colorectal cancer, *EC* esophageal squamous cell carcinoma, *GC* gastric cancer, *NEC* neuroendocrine carcinoma, *NET* neuroendocrine tumor

^a^Site of primary tumor was only collected for patients with NENs. The information of site of primary tumor was not recorded in two patients with NEN

### Safety profiles

In the dose-escalation stage, 16 patients were enrolled. Fifteen of them were included in the DLT evaluable population (1 patient excluded due to other reason). Among them, 1 patient in the 300 mg cohort reported a DLT event, a grade 3 hyperthyroidism which was deemed related to toripalimab. This DLT event started on Day 16 from the first dose, and was recovered on Day 30 with the treatment of the supportive medication and dose interruption of both study treatments.

As of the data cut-off date, 30 patients were included in the safety population. All patients had at least 1 TRAE of any grade. Of the 30 patients, 15 (50%) experienced 1 or more TRAE of grade ≥ 3. The most common (≥ 5% incidence) TRAE of grade ≥ 3 included hypertension [6 patients (20.0%) (1 patient in the 200 mg cohort, 3 patients in the 250 mg cohort, and 2 patients in 300 mg cohort)], transaminase increased [4 patients (13.3%) (1 patient in the 200 mg cohort and 3 patients in the 300 mg cohort)], blood bilirubin increased [4 patients (13.3%) (1 patient in the 250 mg cohort and 3 patients in the 300 mg cohort)], vomiting [2 patients (6.7%) in the 300 mg cohort], asthenia [2 patients (7.1%) in the 300 mg cohort], and anaemia [2 patients (6.7%) (1 patient in the 250 mg cohort and 1 patients in the 300 mg cohort)] (Table 2).

**Table 2:** Summary of treatment-related AEs of any grade occurring in ≥ 20% of total patients or treatment-related grade 3 or worse AEs occurring in ≥ 5% of total patients (safety population)

System organ class preferred term	Surufatinib 200 mg QD + toripalimab 240 mg Q3W (*n* = 6) *n* (%)		Surufatinib 250 mg QD + toripalimab 240 mg Q3W (*n* = 12) *n* (%)		Surufatinib 300 mg QD + toripalimab 240 mg Q3W (*n* = 12) *n* (%)		Total (*n* = 30) *n* (%)	
	Any Grade	≥ Grade 3	Any Grade	≥ Grade 3	Any Grade	≥ Grade 3	Any Grade	≥ Grade 3
Subjects with any treatment-related AEs	6 (100.0)	2 (33.3)	12 (100.0)	4 (33.3)	12 (100.0)	9 (75.0)	30 (100.0)	15 (50.0)
Proteinuria^a^	6 (100.0)	0	9 (75.0)	0	12 (100.0)	0	27 (90.0)	0
Blood bilirubin increased^b^	5 (83.3)	0	9 (75.0)	1 (8.3)	9 (75.0)	3 (25.0)	23 (76.7)	4 (13.3)
Fecal occult blood positive^c^	5 (83.3)	0	9 (75.0)	0	9 (75.0)	0	23 (76.7)	0
Hypertension^d^	5 (83.3)	1 (16.7)	6 (50.0)	3 (25.0)	8 (66.7)	2 (16.7)	19 (63.3)	6 (20.0)
Blood urine present^e^	1 (16.7)	0	7 (58.3)	0	8 (66.7)	0	16 (53.3)	0
White blood cell count decreased	4 (66.7)	0	5 (41.7)	0	6 (50.0)	0	15 (50.0)	0
Hypothyroidism	1 (16.7)	0	4 (33.3)	0	8 (66.7)	0	13 (43.3)	0
Transaminases increased^f^	3 (50.0)	1 (16.7)	3 (25.0)	0	6 (50.0)	3 (25.0)	12 (40.0)	4 (13.3)
Anaemia^g^	0	0	3 (25.0)	1 (8.3)	8 (66.7)	1 (8.3)	11 (36.7)	2 (6.7)
Amylase increased	2 (33.3)	0	5 (41.7)	0	2 (16.7)	1 (8.3)	9 (30.0)	1 (3.3)
Neutrophil count decreased	2 (33.3)	0	2 (16.7)	0	4 (33.3)	0	8 (26.7)	0
Hyperthyroidism	2 (33.3)	0	1 (8.3)	0	4 (33.3)	1 (8.3)	7 (23.3)	1 (3.3)
Blood creatine phosphokinase increased	1 (16.7)	0	1 (8.3)	0	4 (33.3)	1 (8.3)	6 (20.0)	1 (3.3)
Blood creatine phosphokinase MB increased	0	0	1 (8.3)	0	5 (41.7)	0	6 (20.0)	0
Blood urea increased	0	0	3 (25.0)	0	3 (25.0)	0	6 (20.0)	0
Hypertriglyceridaemia^h^	0	0	2 (16.7)	0	4 (33.3)	0	6 (20.0)	0
Diarrhoea	0	0	3 (25.0)	0	3 (25.0)	0	6 (20.0)	0
Vomiting	0	0	1 (8.3)	0	3 (25.0)	2 (16.7)	4 (13.3)	2 (6.7)
Asthenia	1 (16.7)	0	0	0	2 (16.7)	2 (16.7)	3 (10.0)	2 (6.7)

*AE* adverse event

^a^Proteinuria included proteinuria and protein urine present

^b^Blood bilirubin increased included blood bilirubin increased, total bilirubin increased, direct bilirubin increased, indirect bilirubin increased, and bilirubin increased

^c^Fecal occult blood positive included fecal occult blood positive, occult blood positive and occult blood

^d^Hypertension included hypertension and blood pressure increased

^e^Blood urine present included blood urine present and red blood cells urine positive

^f^Transaminases increased included transaminases increased, alanine aminotransferase increased, and aspartate aminotransferase increased

^g^Anaemia included anaemia and haemoglobin decreased

^h^Hypertriglyceridaemia included hypertriglyceridaemia and blood triglycerides increased

Six (20.0%) patients experienced treatment-related serious adverse events (SAEs), 4 of which were observed in the 300 mg cohort [4/12 patients (33.3%)]; the remaining 2 patients [2/12 patients (16.7%)] were in the 250 mg cohort. Treatment-related SAEs included blood bilirubin increased, blood glucose increased, decreased appetite, appendicitis perforated, hyperthyroidism, and gastrointestinal haemorrhage. No fatal treatment-related SAE was reported.

TRAE caused dose interruption or dose reduction in 16 patients (53.3%), with a frequency of 58.3% (7/12 patients) in 300 mg cohort, which is similar with incidence from the other 2 cohorts (50.0% for the 200 and 250 mg cohort, respectively).

There was no TRAE that led to treatment discontinuation in this study.

The incidence of immune-related AEs was 83.3% in the safety population and was comparable among all dose cohorts (66.7% in the 200 mg cohort, 91.7% in the 250 mg cohort, and 83.3% in the 300 mg cohort).

### Pharmacokinetics

The PK parameters of surufatinib were available for 30 patients for Cycle 1 Day 1, 27 patients for Cycle 2 Day 1, and 22 patients for Cycle 3 Day 1. The exposure of surufatinib in terms of area under the concentration–time curve for a dosing interval (AUC_0-τ_) reached steady state on Cycle 2 Day 1 after 200 to 300 mg QD with accumulation ratio of 1.8, 2.0, and 2.1 compared to those on Cycle 1 Day 1, respectively. And the AUC_0-τ_ at steady state increased with the dose ascending from 200 to 300 mg.

The number of patients for the toripalimab PK analysis was 30, 25 and 22 for Cycle 1 Day 1, Cycle 2 Day 1, and Cycle 3 Day 1, respectively. The exposure of toripalimab in terms of AUC_0-τ_ reached steady state on Cycle 2 Day 1 after 240 mg Q3W with accumulation ratio of 1.3–1.5 compared to those on Cycle 1 Day 1. After concomitant administration with surufatinib at different dose levels, the toripalimab exposure (AUC_0-τ_) was similar, which may indicate no obvious impact of surufatinib administration on the toripalimab exposure in patients.

The major PK parameters of surufatinib and toripalimab are listed in Supplementary Table S2.

### Preliminary effectiveness outcomes

**Fig. 1:**
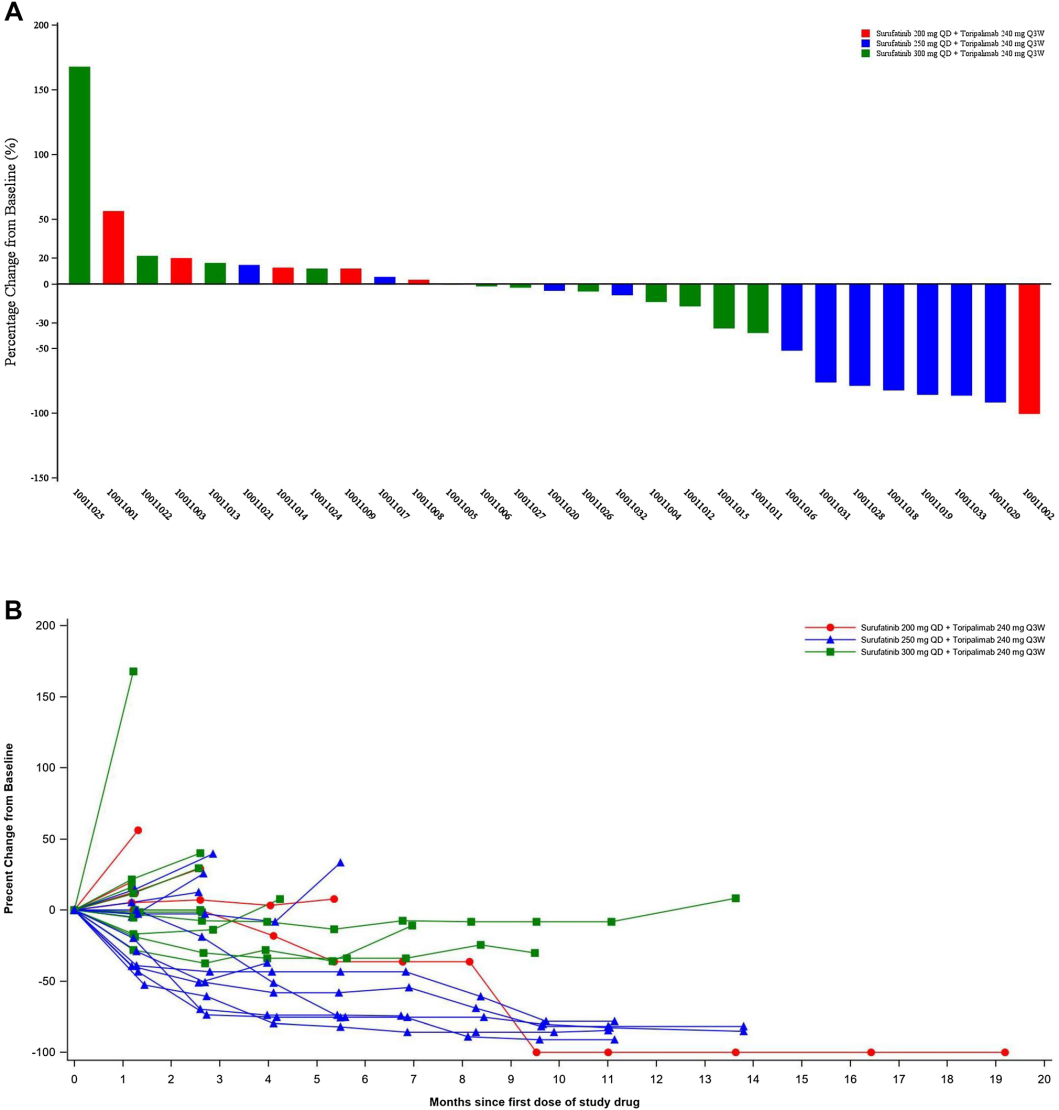

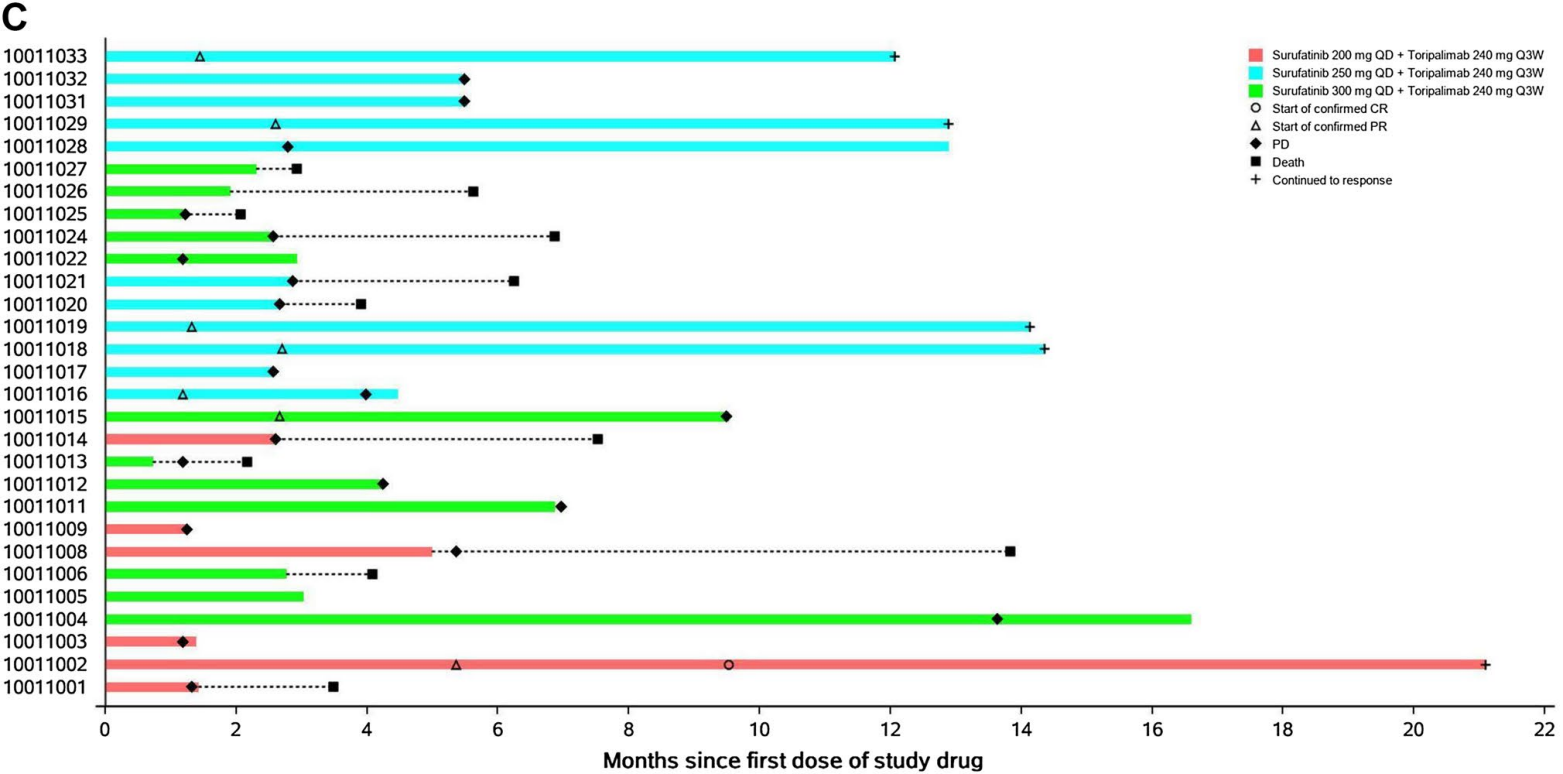
**a** Waterfall plot—best percent change from baseline in sum of target lesion diameters (efficacy evaluable population)*; **b** spider plot—percent change from baseline in sum of target lesion diameters over time (efficacy evaluable population)*; **c** swimmer plot—response pattern of each subject (efficacy evaluable population)*. *Only assessments prior to initial of a new anti-cancer therapy included in the analysis. *CR* complete response, *PR* partial response, *SD* stable disease, *PD* progressive disease

Twenty-nine of 30 enrolled patients were evaluable for tumor response. By the data cutoff, there were 1 patient with a confirmed complete response (CR), 6 patients with confirmed partial responses (PRs), and 16 patients with stable disease (SD) as their best overall response (BOR) among the evaluable patients (Fig. 1a). Most of the patients with confirmed PRs (5/6 patients) were from the 250 mg cohort, except for 1 patient in the 300 mg cohort. The overall ORR and DCR were 24.1% and 79.3%, respectively. The ORR in the 200 mg, 250 mg, and 300 mg cohorts was 16.7%, 45.5%, and 8.3%, respectively; the DCR was 50.0%, 100%, and 75.0%, respectively (Table 3).

**Table 3:** Tumor response of surufatinib plus toripalimab (efficacy evaluable population)

	Surufatinib 200 mg QD + toripalimab 240 mg Q3W (*n* = 6) *n* (%)	Surufatinib 250 mg QD + toripalimab 240 mg Q3W (*n* = 11) *n* (%)	Surufatinib 300 mg QD + toripalimab 240 mg Q3W (*n* = 12) *n* (%)	Total (*n* = 29) *n* (%)
Confirmed best overall response (based on RECIST v1.1)				
CR	1 (16.7)	0 (0.0)	0 (0.0)	1 (3.4)
PR	0 (0.0)	5 (45.5)	1 (8.3)	6 (20.7)
SD	2 (33.3)	6 (54.5)	8 (66.7)	16 (55.2)
PD	3 (50.0)	0 (0.0)	3 (25.0)	6 (20.7)
ORR^a^	1 (16.7)	5 (45.5)	1 (8.3)	7 (24.1)
95% CI of ORR	0.4–64.1	16.7–76.6	0.2–38.5	10.3–43.5
DCR^b^	3 (50.0)	11 (100.0)	9 (75.0)	23 (79.3)
95% CI of DCR	11.8–88.2	71.5–100.0	42.8–94.5	60.3–92.0

*CR* complete response, *PR* partial response, *SD* stable disease, *PD* progressive disease, *ORR* objective response rate, *DCR* disease control rate, *CI* confidence interval, *RECIST* Response Evaluation Criteria in Solid Tumors

^a^ORR was defined as the proportion of patients with confirmed best overall response of CR or PR

^b^DCR was defined as the proportion of patients with best overall response of CR or PR or SD

Decrease in target lesion size was observed in 17 patients (59.3%), most of whom were in the 250 mg cohort (9 patients). There were 1 patient with a confirmed CR and 4 patients with confirmed PRs with response trends that were continuing at the cut-off date (Fig. 1b, c).

Five of the 21 evaluable NENs patients achieved an objective response, including 1 confirmed CR in patient with NEC, 2 confirmed PRs in patients with NEC, and 2 confirmed PRs in patients with NET G2. For the remaining NEN patients, 11 patients had SD, including 2 patients in the 200 mg cohort, 3 patients in the 250 mg cohort, and 6 patients in the 300 mg cohort.

A response of SD or better was seen in all 4 CRC patients, 1 patient with a confirmed PR (MSI-H, 250 mg cohort), and 3 patients with SD (all MSS, 2 patients in the 250 mg cohort, 1 patient in the 300 mg cohort).

At the data cutoff, tumor progression or death was observed in 22 patients (maturity of PFS data, 73.3%), and overall median PFS (mPFS) was 4.0 months (95% CI 2.6‒5.5). The mPFS was 2.0 months (95% CI 1.2‒not reached) for the 200 mg cohort, 5.5 months (95% CI 2.7‒not reached) for the 250 mg cohort, and 4.1 months (95% CI 1.2‒9.5) for the 300 mg cohort (Supplementary Figure S1). The overall 6 month PFS rate was 30.3% (95% CI 14.5‒47.9%). Median DoR, TTR, and OS were not reached at the cut-off date, and the overall 6-month OS rate was 76.7% (95% CI 57.2‒88.1%).

### Biomarker analysis

Tumor tissues from 27 patients at baseline were available for PD-L1 expression analysis. Among the 14 PD-L1-positive patients, 1/14 (7.1%) patient achieved a confirmed CR and 4/14 patients (28.6%) achieved confirmed PRs, while 6/14 (42.9%) reported SD. In the 13 patients with negative PD-L1 status, 10/13 (76.9%) achieved their BOR as SD or better, including 2/13 patients (15.4%) with confirmed PRs, and 8/13 patients (61.5%) with SD.

## Discussion

The results of this phase 1, open-label study showed that the combination treatment of surufatinib and toripalimab was reasonably well tolerated. There was no new safety signal noted in this study.

Most TRAEs in this study were mild (grade 1 or 2), and were manageable with supportive care medications and dose adjustments. No treatment-related discontinuation or death was identified. The incidence of TRAEs of grade ≥ 3 in surufatinib and toripalimab combination was 50%, with hypertension being the most common TRAE. The grade ≥ 3 toxicity profile observed in this study was consistent with previously reported safety data for surufatinib or toripalimab monotherapies (Xu et al. 2019; Chen et al. 2020; Wang et al. 2019; Yang et al. 2020). Moreover, the frequencies of grade 3 or worse TRAEs were comparable or slightly worse than those reported in other VEGF/VEGFR inhibitor plus PD-1/PD-L1 antibody combinations. For example, in one phase 1b and one phase 3 study of axitinib combined with pembrolizumab for the treatment of patients with advanced renal cell cancer, the incidences of grade 3 or worse TRAEs were 65% and 67%, respectively, with hypertension as the most common TRAEs of grade ≥ 3 (Atkins et al. 2018; Powles et al. 2020). In another phase 3 study of axitinib plus avelumab for advanced renal cell carcinoma, the incidence of grade 3 or worse TRAE was 56.7% (Motzer et al. 2019).

Proteinuria, hypertension, and abnormal liver function are common in surufatinib monotherapy (Xu et al. 2019; Chen et al. 2020); the frequencies of these adverse events in this study were consistent with previous findings. Common immune-related toxicity related to toripalimab in its combination with axitinib included diarrhea (60.6%), hypothyroidism (51.5%), alanine aminotransferase increased (42.4%), rash (36.4%), and aspartate aminotransferase increased (33.3%) (Sheng et al. 2019). Similar immune-related toxicity profile was identified in this study, with the exception of rash, whose incidence was 13.3% and clearly lower.

Safety data suggested that 200 mg or 250 mg surufatinib plus toripalimab appear to have the more tolerable toxicity profile. Patients in 300 mg cohort experienced more grade ≥ 3 TRAEs, treatment-related SAEs, and immune-related AEs. In addition, one DLT was observed in the 300 mg cohort.

We noted that among the 29 patients evaluable for efficacy, the majority of the patients with confirmed PRs (5/6) were in 250 mg cohort, which was accompanied by the highest ORR (45.5%). In spite of the efficacy differences among the different dosage groups, which might due to multiple factors such as different tumor types and baseline characteristics, the efficacy results indicated that 250 mg QD surufatinib was the most effective dose in this combination therapy. Based on the observed promising preliminary efficacy results, as well as the favorable safety profile for 250 mg cohort, the RP2D was determined to be 250 mg QD surufatinib plus 240 mg Q3W toripalimab.

Previously, in 2 phase 3 trials of surufatinib in advanced well-differentiated NET, ORRs were 19.2% (95% CI 12.2‒28.1) and 10.3% (95% CI 5.6‒17.0) in the pancreatic and extrapancreatic NET, respectively, and DCRs were 80.8% (95% CI 71.9‒87.8) and 86.5% (95% CI 79.3‒91.9), respectively (Xu et al. 2020a, b). In this study, among the 21 NEN patients with evaluable tumor assessment (more than half are NECs), the ORR and DCR was 23.8% and 76.2%, respectively. At the RP2D (250 mg cohort), the ORR and DCR in patients with NEN was 50% and 100%, respectively. In our previous work of evaluating the safety and efficacy of using toripalimab in NEN patients (Ki-67 ≥ 10%) after failure of first line therapy, the ORR and DCR were 20% and 35%, respectively (Lu et al. 2020). In this current study, the combination of surufatinib and toripalimab in the RP2D cohort achieved a higher response rate of 50%, compared with results from the respective single-agent studies, which indicates a potential synergistic effect between these two agents.

Particularly, among the 13 evaluable patients with advanced NEC, there was 1 patient with BOR confirmed as a CR, and 2 confirmed as PRs. The ORR in advanced NEC population was 23.1%, with mPFS of 4.0 months, and mOS of 7.5 months, which are comparable or numerically better with the efficacy results from the second-line chemotherapy in treatment of advanced NEC (mPFS of 2.3 months and mOS of 6 months, respectively) (McGarrah et al. 2018).

During preclinical phase, the mechanism of this combination was well studied. Tumor angiogenesis could be promoted by many factors, such as VEGF, basic fibroblast growth factor (bFGF), and platelet-derived endothelial growth factor (PDGF) (Carmeliet and Jain 2020). Angiogenesis could induce an immunosuppressive tumor microenvironment and was found to be closely related with tumor immune escape (Buckanovich et al. 2008). The anti-VEGFR and FGFR treatment, such as surufatinib, could suppress tumor angiogenesis and overcome the resistance to VEGF/VEGFR inhibition, thus enhance the effect of immune check point inhibitors (Zhou et al. 2017). In addition to the inhibition of VEGFR and FGFR, surufatinib could also inhibit CSF-1R and CSF-1R is involved in tissue macrophage development and maintenance (Stanley et al. 1978). By inhibiting CSF-1R, surufatinib could significantly decrease CSF-1R positive M2 TAM infiltration in tumor tissues, and increase the infiltration of M1-TAM (iNOS +) and CD8 + T cell, which result in enhancement of immune response (Zhou et al. 2017). These findings indicate that surufatinib could simultaneously block tumor angiogenesis and modulate cancer immunity, which might explain the improved efficacy of the combination of surufatinib with toripalimab.

For the predictive biomarker exploration, we tested PD-L1-positive status in enrolled patients. In previous published results, the ORR of toripalimab and axitinib was higher in PD-L1 positive (≥ 1%) patients (Sheng et al. 2019), and PD-L1 expression seemed to be a good predictive marker for toripalimab (Lu et al. 2020). However, in our study, we observed potential clinical benefit for the entire patient population, regardless of PD-L1 expression. The ORR and DCR were comparable in PD-L1-positive and PD-L1-negative population. This might be due to the dual angio-immuno approach of surufatinib, by inhibiting a trifecta of cancer-promoting receptors (Xu et al. 2019; Chen et al. 2020). Given the small sample size employed in our study, the potential for benefiting all patients regardless of PD-L1 expression requires confirmation in a larger randomized study.

The limitation of this phase 1 study includes the small sample size represented in the patient population. A phase 2 trial of surufatinib plus toripalimab (NCT04169672) has been initiated to further evaluate the efficacy and safety profile of this combination in advanced solid tumors, which mainly include NEN, biliary tract cancer, gastric cancer, thyroid cancer, small cell lung cancer, soft-tissue sarcoma, endometrial carcinoma, and esophageal squamous cell carcinoma.

## Conclusion

In summary, surufatinib plus toripalimab was well tolerated with no unexpected safety signals observed and showed preliminary anti-tumor activity in patients with advanced solid tumor, particularly in NEN patients. This combination warrants further evaluations in clinical trials.

## Supplementary Information

Below is the link to the electronic supplementary material.

Supplementary file1 Supplementary Figure S1 Progression-free survival (Kaplan-Meier plot). Abbreviations: CI, confidence interval; PFS, progression-free survival; SUR, surufatinib; TOR, toripalimab; Qd, once daily; Q3W, every 3 weeks. (TIF 4994 KB)

Supplementary file2 (DOCX 30 KB)

## Data Availability

The datasets generated during and/or analysed during the current study are available from the corresponding author on reasonable request.
