# Supplementary figures and images for: Surufatinib plus toripalimab in patients with advanced solid tumors: a single-arm, open-label, phase 1 trial
Source: J Cancer Res Clin Oncol. 2022 Feb 15;149(2):779–89. doi: 10.1007/s00432-021-03898-8 (PMC9931771; doi:10.1007/s00432-021-03898-8)

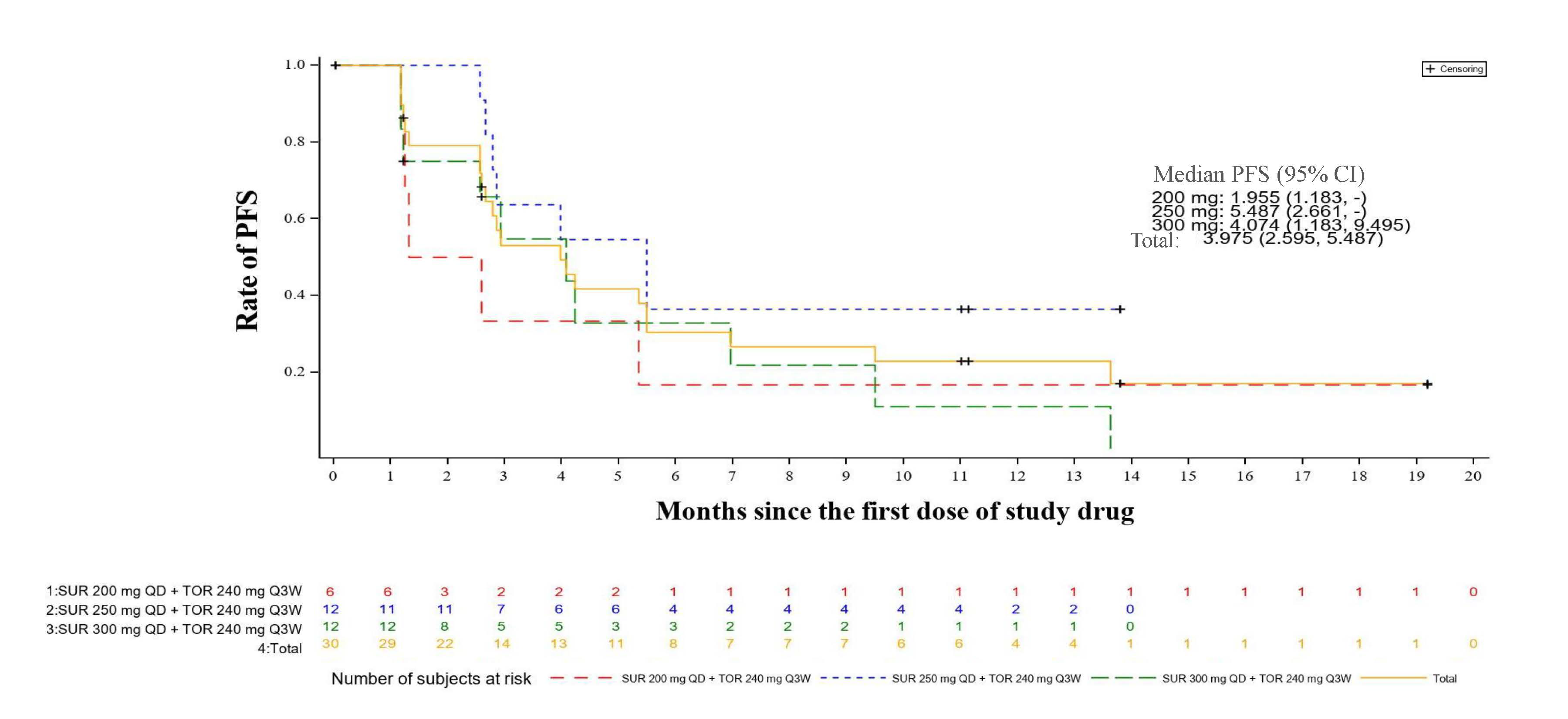

Supplement: Supplementary file 1 — Supplementary file1 Supplementary Figure S1 Progression-free survival (Kaplan-Meier plot). Abbreviations: CI, confidence interval; PFS, progression-free survival; SUR, surufatinib; TOR, toripalimab; Qd, once daily; Q3W, every 3 weeks. (TIF 4994 KB) [file 432_2021_3898_MOESM1_ESM.tif]
