# Supplementary material for: Surufatinib plus toripalimab in patients with advanced solid tumors: a single-arm, open-label, phase 1 trial
Source: J Cancer Res Clin Oncol. 2022 Feb 15;149(2):779–89. doi: 10.1007/s00432-021-03898-8 (PMC9931771; doi:10.1007/s00432-021-03898-8)
Supplement: Supplementary file 2 — Supplementary file2 (DOCX 30 KB) [file 432_2021_3898_MOESM2_ESM.docx]

**Surufatinib plus Toripalimab in Patients with Advanced Solid Tumors: a Single-Arm, Open-Label, Phase 1 Trial**

**Supplementary Information**

**Journal Name:**

Journal of cancer research and clinical oncology

**Authors:**

Yanshuo Cao^1*^, Ming Lu^1*^, Yu Sun^2^, Jifang Gong^1^, Jie Li^1^, Zhihao Lu^1^, Jian Li^1^, Xiaotian Zhang^1^, Yan Li^1^, Zhi Peng^1^, Jun Zhou^1^, Xicheng Wang^1^, Lin Shen^1,3^

* Y. Cao and M. Lu contributed equally to this work.

**Corresponding Author:**

Lin Shen, Peking University Cancer Hospital & Institute, Fu-Cheng Road 52, Hai-Dian District, Beijing 100142, China.

Table S1 Inclusion and exclusion criteria

| **Inclusion criteria**^1^ |
| --- |
| 1. Patients who are fully informed about the study and voluntarily sign the ICF (prior to the implementation of any specific procedure for the trial); |
| 2. 18–75 years old (inclusive); |
| 3. Patients with histological or cytological diagnosis of inoperable or metastatic advanced solid tumors (mainly gastrointestinal neoplasms such as neuroendocrine neoplasm, hepatocellular carcinoma, and gastric cancer); |
| 4. Patients who have failed standard treatment (due to disease progression after the treatment or intolerable toxicity or side effects during the treatment) or have not responded to currently available therapies; |
| 5. ECOG performance status of 0 or 1; |
| 6. For patients with advanced primary cancer of liver, the Child-Pugh score shall be A or better B (≤ 7 points); |
| 7. Patients identified with measurable lesions that meet the criteria for solid tumor efficacy evaluation (RECIST 1.1); if the lesion previously treated with local therapy (radiation therapy, ablation, vascular intervention, etc.) is the only lesion, a valid imaging evidence for the progression of the lesion will be required; |
| 8. Patients who have adequate organ function of bone marrow, liver and kidney, as well as laboratory test results within seven days before the first dose that meet the following criteria (neither blood transfusion, blood products, nor corrections with granulocyte colony stimulating factor or other hematopoietic stimulate factor are taken within 14 days before laboratory test):  • ANC ≥ 1.5 × 10^9^/L, PLT ≥ 100 × 10^9^/L, and hemoglobin ≥ 90 g/L;  • Serum bilirubin total ≤ 1.5 × ULN;  • In the absence of metastases to liver, ALT and AST ≤ 2.0 × ULN; in the presence of metastases to liver, ALT and AST ≤ 3 × ULN;  • Serum creatinine ≤ 1.5 × ULN and creatinine clearance ≥ 50 mL/min (calculated according to the Cockcroft-Gault formula);  • Urinalysis shows urinary protein of < 2+, otherwise 24-hour urinary protein of < 1 g;  • INR ≤ 1.5 and APTT ≤ 1.5 × ULN; |
| 9. Expected survival of ≥ 12 weeks; |
| 10. For women with child-bearing potential, the results of a serum or urine pregnancy test must be negative within 7 days of the first dose. Fertile male or female patients shall volunteer to use effective contraceptive methods, such as double barrier contraception, condoms, oral or injected contraceptives, and intra-uterine contraceptive devices, during the study period and within 90 days after the last dosing of the investigational drug. All female patients will be considered fertile unless they have had natural menopause, or artificial menopause or sterilization (such as hysterectomy, bilateral adnexectomy or ovarian radiation). |
| **Exclusion Criteria^2^** |
| 1. Toxicity associated with previous antineoplastic treatment that has not recovered to ≤ CTCAE grade 1, excluding alopecia or peripheral neurotoxicity which is ≤ CTCAE grade 2 caused by oxaliplatin; |
| 2. Other malignant tumors in the past 5 years, except basal/squamous cell carcinoma or carcinoma in situ of the cervix that has been controlled adequately; |
| 3. Currently suffering from metastasis in the CNS or previous brain metastasis; |
| 4. Having received a systemic anti-tumor therapy that has been approved or in development within 2 weeks prior to the first dose, including chemotherapy, bio-immunotherapy, targeted therapy, and treatment with traditional Chinese medicines (if the instructions of the traditional Chinese medicines specify definite indications for anti-tumor therapy, the patient may be enrolled after an 1-week washout period prior to the first dose); |
| 5. Having received a radical radiation therapy within 4 weeks before the first dose (including more than 25% radiation therapy in bone marrow); |
| 6. Having received any treatment with anti-PD-1 antibody, anti-PD-L1 antibody, anti-PD-L2 antibody or anti-CTLA-4 antibody (or any other antibodies acting on T cell co-stimulation or checkpoint pathway) or surufatinib; |
| 7. Having received a systemic treatment with corticosteroids (prednisone at a dose of > 10 mg/d or other hormones with equivalent efficacy) or other immunosuppressive agents within 2 weeks prior to the first dose; nasal spray, aspiration or other topical hormones (i.e., prednisone at a dose of no more than 10 mg/d, or other glucocorticoids at an equivalent efficacy dose) are allowed; |
| 8. Having any history of active autoimmune disorder or autoimmune disorder, including but not limited to interstitial pneumonitis, uveitis, inflammatory bowel disorder, hepatitis, hypophysitis, vasculitis, and systemic lupus erythematosus (patients with vitiligo and psoriasis requiring no systemic treatment in the past 2 years or thyroid function decreased that can be controlled by hormone replacement therapy only, and patients with type I diabetes who only need insulin replacement therapy can be enrolled); |
| 9. Having received any live or attenuated vaccine within 4 weeks prior to the first dose, or planning to do so during the study |
| 10. Having undergone major surgery within 4 weeks prior to the first dose (for the definition of major surgery, refer to the level 3 and level 4 operations specified in the "Management Regulations for Clinical Application of Medical Technology" effective on May 1, 2009), or with unhealed wounds, ulcers, or fractures; |
| 11. With uncontrolled malignant hydrothorax, ascites or pericardial effusion, which cannot be effectively controlled by diuretics or puncture as judged by the investigator |
| 12. With hypertension that is uncontrollable by medications, defined as: systolic blood pressure ≥ 140 mmHg and/or diastolic blood pressure ≥ 90 mmHg |
| 13. With any disease or condition affecting drug absorption, or patients cannot be treated via oral drug administration; |
| 14. Having received a potent inducer or inhibitor of CYP3A4 within 2 weeks prior to the first dose, or needing to continue the treatment with such medications during the study; |
| 15. Currently suffering from active gastric and duodenal ulcer, ulcerative colitis and other gastrointestinal diseases, active bleeding caused by unresected tumors, or other conditions that may lead to gastrointestinal bleeding or perforation as judged by the investigator; |
| 16. With evidence or history of apparent bleeding tendency within 2 months prior to the first dose (bleeding volume > 30 mL over 2 months, with hematemesis, melena, or hematochezia), hemoptysis (> 5 mL of fresh blood over 4 weeks), or history of thromboembolism within the past 12 months (including stroke and/or transient ischemic attack); |
| 17. With clinically significant cardiovascular diseases, including but not limited to: acute myocardial infarction, severe/unstable angina pectoris or coronary artery bypass grafting within 6 months prior to the first dose; congestive heart failure of NYHA grade > 2; and LVEF < 50%; |
| 18. Clinically significant serious electrolyte abnormalities as determined by the investigator; |
| 19. With active infection or fever of unknown origin (body temperature > 38.5 °C) during the screening or prior to the first dose; |
| 20. Having active TB and receiving anti-tuberculosis treatment or having received anti-tuberculosis treatment within 1 year prior to the first dose; |
| 21. With a previous or current history of pulmonary fibrosis, interstitial pneumonitis, pneumoconiosis, radiation pneumonitis, drug-related pneumonitis, and other severe impairment of lung function that may interfere with the assay and treatment of suspected drug-related pulmonary toxicity; radiation pneumonitis in the radiotherapy area is allowed |
| 22. Known infection of HIV; |
| 23. Known history of clinically significant liver diseases, including viral hepatitis [for known carriers of HBV, the presence of active HBV infection, i.e., positive HBV DNA (> 1 × 103 copies/mL or > 200 IU/mL) must be excluded; subjects who are positive to hepatitis B virus core antibody (HbcAb) and negative to hepatitis B virus surface antigen (HBsAg) can be enrolled into this study, but must be tested for HBV DNA before first dose; known infection of HCV with positive HCV RNA (> 1 × 103 copies/mL)], or other hepatitis or clinically significant cirrhosis; |
| 24. Having participated in clinical trials of other investigational drugs which have not been approved or marketed in China and received corresponding treatment within 4 weeks prior to the first dose; |
| 25. Pregnant (positive pregnancy test before dosing) or breast-feeding women; |
| 26. Known to be allergic to any components in toripalimab or surufatinib, or with a history of severe allergies to any other monoclonal antibodies; |
| 27. Any other diseases with clinically significant metabolic abnormalities (such as uncontrollable diabetes, thyroid function abnormal), abnormal physical observations or abnormal laboratory findings, which are judged by the investigator as evidence that the patient has a disease or condition that is unsuitable for the investigational drug (e.g., epileptic seizures requiring treatment), or that would interfere with the interpretation of the study results, or that may put the patient at a high risk. |

ALT, alanine transaminase; ANC, absolute neutrophil count; APTT, activated partial thromboplastin time; AST, Aspartate transaminase; CTCAE, common terminology criteria for adverse events; CTLA-4, cytotoxic T-lymphocyte-associated protein 4; ECOG, European Cooperative Oncology Group; HBV, hepatitis B virus; HCV, hepatitis C virus; HIV, human immunodeficiency virus; ICF, informed consent form; INR, international normalized ratio; LVEF, left ventricular ejection fraction; NYHA, New York Heart Association; RECIST, response evaluation criteria in solid tumors; PD-1; programmed death-1; PD-L1, programmed death ligand-1; PLT, blood platelet; ULN, upper limit of normal;

^1^Patients meet any of the following criteria must be excluded from this study.

^2^Patients who meet any of the following criteria must be excluded from this study.

Table S2 PK parameters of surufatinib and toripalimab (PK population)

| **Surufatinib** | | | | | | | | | | | |
| --- | --- | --- | --- | --- | --- | --- | --- | --- | --- | --- | --- |
| Dose (mg) | | Day | N | C_max_ (ng/mL) | T_max_ (h) | | AUC_0-τ_ (h×ng/mL) | t_1/2_ (h) | | CL/F (L/h) | Vd/F（L） |
| Surufatinib | Toripalimab |  |  | Geometric mean (CV%) | Median | min, max | Geometric mean (CV%) | Median | min, max | Geometric mean (CV%) | Geometric mean (CV%) |
| 200 | 240 | C1D1 | 6 | 277 (27.9) | 1.98 | 1.08, 4.00 | 1845 (8.53)^1^ | 4.49 ^1^ | 3.75 ,35.1 | 89.5 (23.9) ^1^ | 925.8 (97.9) ^1^ |
| 250 | 240 | C1D1 | 12 | 289 (58.3) | 1.60 | 1.00, 4.00 | 1845 (54.5) | 11.1 | 7.12 ,12.7 | 110 (34.6) | 1660 (31.1) |
| 300 | 240 | C1D1 | 12 | 228 (46.3) | 2.00 | 1.10, 4.00 | 1878 (72.4)^1^ | 8.12 ^1^ | 4.10 ,16.7 | 134 (30.7) ^1^ | 1615 (59.4) ^1^ |
| 200 | 240 | C2D1 | 6 | 268 (55.0) | 2.97 | 1.90, 4.00 | 3013 (42.6)^2^ | 13.1 ^4^ | 12.20 ,16.6 | 66.4 (38.3) ^2^ | 66.4 (38.3)^4^ |
| 250 | 240 | C2D1 | 11 | 407 (71.4) | 2.00 | 1.00, 4.03 | 3610 (68.0) | 11.9 | 8.54 ,17.2 | 69.2 (45.7) | 69.2 (45.7) |
| 300 | 240 | C2D1 | 10 | 281 (34.0) | 2.97 | 1.00, 7.93 | 3668 (30.4)^2^ | 14.4 ^4^ | 10.79 ,19.6 | 81.8 (27.4) ^2^ | 81.8 (27.4)^4^ |
| 200 | 240 | C3D1 | 3 | 239 (53.6) | 1.98 | 1.98, 4.00 | 2298 (24.3)^3^ | 10.1 ^3^ | 4.86 ,15.3 | 87.0 (24.3) ^3^ | 1084 (89.7)^3^ |
| 250 | 240 | C3D1 | 9 | 310 (58.8) | 1.93 | 1.00, 4.00 | 2398 (52.3)^3^ | 15.6 ^3^ | 12.85 ,40.6 | 104 (45.0)^3^ | 2538 (81.0)^3^ |
| 300 | 240 | C3D1 | 10 | 361 (31.7) | 2.00 | 1.90, 4.10 | 3496 (30.1) | 12.2 | 6.04 ,28.4 | 84.3 (34.9) | 1506 (47.7) |
| **Toripalimab** | | | | | | | | | | | |
| Dose (mg) | | Day | N | C_max_ (µg/mL) | T_max_ (h) | | AUC_0-τ_ (h×µg/mL) | t_1/2_ (h) | | CL (mL/h) | Vd/F（L） |
| Surufatinib | Toripalimab |  |  | Geometric mean (CV%) | Median | min, max | Geometric mean (CV%) | Median | min, max | Geometric mean (CV%) | Geometric mean (CV%) |
| 200 | 240 | C1D1 | 6 | 78.1 (15.2) | 1.96 | 0.78, 6.63 | 15925 (22.8) | 231 | 196 ,444 | 11.1 (30.8) | 4073 (16.9) |
| 250 | 240 | C1D1 | 12 | 71.4 (21.6) | 1.85 | 1.03, 24.4 | 14142 (25.6) | 237 | 168 ,311 | 13.0 (32.9) | 4430 (21.5) |
| 300 | 240 | C1D1 | 12 | 81.2 (24.5) | 1.12 | 0.70, 6.78 | 16458 (25.0) | 234 | 170 ,370 | 11.0 (22.1) | 3868 (25.4) |
| 200 | 240 | C2D1 | 4 | 96.2 (17.9) | 2.63 | 1.32, 6.63 | 21512 (26.4) | 320 | 276 ,444 | 11.2 (24.3) | 5389 (19.2) |
| 250 | 240 | C2D1 | 11 | 83.8 (24.5) | 1.13 | 0.62, 6.68 | 17176 (31.5) | 259 | 155 ,357 | 14.0 (37.2) | 4783 (31.5) |
| 300 | 240 | C2D1 | 10 | 98.1 (27.5) | 1.26 | 0.63, 24.2 | 20660 (33.7) | 246 | 164 ,319 | 11.6 (27.6) | 4011 (24.5) |
| 200 | 240 | C3D1 | 3 | 110 (29.0) | 1.45 | 0.98, 2.72 | 24626 (34.9) | 390 | 194 ,459 | 9.75 (29.6) | 4587 (44.7) |
| 250 | 240 | C3D1 | 10 | 97.4 (25.0) | 1.87 | 0.58, 24.2 | 20157 (39.5) | 246 | 69.1 ,367 | 11.9 (52.0) | 3839 (30.3) |
| 300 | 240 | C3D1 | 9 | 103 (33.2) | 1.17 | 0.72, 24.2 | 20063 (46.6) | 241 | 151 ,429 | 12.0 (35.8) | 4068 (25.0) |

C: Cycle; D: Day; PK, pharmacokinetics; C_max_, maximum observed concentration; T_max_, time to C_max_; AUC_0-τ_, area under the concentration-time curve for a dosing interval; CV, coefficient of variation.

^1^N for AUC_0-τ_ in C1D1 was 4 in 200 mg cohort and 10 in 300 mg cohort.

^2^N for AUC_0-τ_ and CL/F in C2D1 was 3 in 200 mg cohort and 9 in 300 mg cohort.

^3^N for AUC_0-τ_ in C3D1 was 2 in 200 mg cohort and 8 in 250 mg cohort.

^4^N for t_1/2_, and Vd/F in C1D1 was 3 in 200 mg cohort and 8 in 300 mg cohort.
